# 2024 Editors’ Highlights

**DOI:** 10.1038/s42004-024-01402-0

**Published:** 2025-01-06

## Abstract

We are pleased to present our 2024 Editors’ Highlights Collection, featuring some of our favorite content published in *Communications Chemistry* in 2024. Here, we highlight why each paper was selected by our Editors.

Editorial Board Member Dr Teodoro Laino highlights *Automatic feature engineering for catalyst design using small data without prior knowledge of target catalysis* by Toshiaki Taniike et al. (10.1038/s42004-023-01086-y). “This study introduces an automatic feature engineering (AFE) technique to address data scarcity in catalyst informatics. By generating and selecting high-order features without prior assumptions, the authors achieve accurate predictions across diverse catalytic systems. The integration of AFE with active learning further exemplifies the potential of machine-driven approaches to streamline catalyst design and discovery, advancing data-driven research for complex materials.”

Editorial Board Member Prof. Kristin Wustholz highlights *A spectroscopic test suggests that fragment ion structure annotations in MS/MS libraries are frequently incorrect* by Lara van Tetering et al. (10.1038/s42004-024-01112-7). “Although tandem mass spectrometry (MS/MS, MS2) is a powerful tool for structural elucidation beyond the chemical formula, users often rely on in silico generated MS2 spectral libraries to determine structure. The team evaluates the fidelity of this approach by comparing predicted structures against structures experimentally established by infrared ion spectroscopy (IRIS). The authors demonstrate that virtually all structure annotations in three major in silico MS2 libraries are incorrect and caution readers against their use.”

Editorial Board Member Prof. Ga-lai Law highlights *A generative artificial intelligence framework based on a molecular diffusion model for the design of metal-organic frameworks for carbon capture* by Hyun Park et al. (10.1038/s42004-023-01090-2). “The team introduces GHP-MOFassemble, an AI framework utilizing a diffusion model to design metal-organic frameworks (MOFs) for enhanced carbon capture. By generating unique MOF linkers assembled with metal nodes, the system can be used to accelerates the discovery of high-capacity CO_2_ adsorption materials. The model, by simulations and neural networks, identifies top-performing MOFs with CO_2_ capacities surpassing 2 m mol g − 1, which is higher than 96.9% of structures in the hypothetical MOF dataset.”

Editorial Board Member Dr André Dallmann highlights *Interactions between the protein barnase and co-solutes studied by NMR* by Clare Trevitt et al. (10.1038/s42004-024-01127-0). “The authors give a very unique insight into probably one of the most understudied yet very important subjects of our time – that is protein stability and solubility in solution. They applied NMR spectroscopy on the model protein barnase to study the effects of anions as well as osmolytes on its stability. They were not only able to unify varying differing models (Hofmeister and reverse Hofmeister series) into one, but even more importantly were able to explain the models based on the thermodynamic properties of the interaction of protein and ions/osmolytes. Anyone working in the field of structural biology trying to keep their target protein alive and afloat as long as possible will recognize this paper’s crucial impact on their work immediately.”

Editorial Board Member Dr Christian Agatemor highlights *Body odor samples from infants and post-pubertal children differ in their volatile profiles* by Diana Owsienko et al. (10.1038/s42004-024-01131-4). “Body odor, a chemo-signal that influences interpersonal communication, changes during development. Yet the molecular basis underpinning this change, specifically in infant to post-pubertal children, remains unclear. The authors show that post-pubertal children have a higher odor dilution factor of carboxylic acids and a higher concentration of squalene than infants. The study indicates that sexual maturation coincides with changes in body odor chemical composition, but whether this change contributes to the olfactory perception of infants versus post-pubertal children needs to be demonstrated.”

Editorial Board Member Dr Alina Sekretareva highlights *Quantification and description of photothermal heating effects in plasmon-assisted electrochemistry* by Md. Al-Amin et al. (10.1038/s42004-024-01157-8). “This paper demonstrates the significant yet often overlooked role of plasmonic photothermal heating in electrochemical processes. Through a combination of cyclic voltammetry and finite-element simulations, the authors demonstrate that even a tiny temperature gradient of just 1 K between the electrode surface and bulk electrolyte can substantially boost current density. These findings provide new insights into the thermal effects driving plasmon-assisted electrochemistry.”

Editorial Board Member Dr David Nelson highlights *A protocol for controlled reactivity shift in the 2,2-difluorovinyl motif used for selective S–*^*18*^*F and C–*^*18*^*F bond formation* by Mudasir Maqbool et al. (10.1038/s42004-024-01132-3). “The incorporation of the short-lived ^18^F isotope into organic molecules is highly challenging, but essential for many areas of chemistry, biology, and medicine. This study discloses an effective way to introduce [^18^F]trifluoroethyl groups directly into molecules, or to prepare [^18^F]trifluoroethyl tosylate (in 6 minutes) that can then be used to prepare [^18^F]trifluoroethyl piperidines and piperazines in one subsequent step.”

Senior Editor Dr Huijuan Guo highlights *A facile DNA coacervate platform for engineering wetting, engulfment, fusion and transient behavior* by Wei Liu et al. (10.1038/s42004-024-01185-4). “In this contribution, the authors describe a simple and versatile platform for programmable DNA coacervates, by using long single-stranded DNA homopolymer in combination with a series of palindromic binders. They demonstrate assembly/disassembly switching of coacervates, as well as engineering wetting, clustering, engulfment, and merging of the distinct coacervate droplets. The facile tunability and options of functionalization enable fundamental studies into coacervate dynamics and structures.”

Editorial Board Member Dr Marina Šekutor highlights *Precision engineering of nano-assemblies in superfluid helium by the use of van der Waals forces* by G. Topcu et al. (10.1038/s42004-024-01203-5). “The authors describe a templating approach for growing nanoparticle assemblies in superfluid helium nanodroplets, achieved by using weak van der Waals interactions between gold and the introduced organic molecules. The ability to precisely control nanoparticle growth with natural self-organization principles is impactful for plasmonic and photonic device miniaturization that strives towards a sub-nm precision.”

Editorial Board Member Dr Indranath Chakraborty highlights *Globular pattern formation of hierarchical ceria nanoarchitectures* by Noboru Aoyagi et al. (10.1038/s42004-024-01199-y). “This recent study reports in-situ real-time observation of hierarchical CeO_2_ nanoarchitectures formed in a droplet which has substantial implications for colloidal chemistry and non-equilibrium physics.”

Editorial Board Member Prof. Andy Wilson highlights *Design of a covalent protein-protein interaction inhibitor of SRPKs to suppress angiogenesis and invasion of cancer cells* by Gongli Cai et al. (10.1038/s42004-024-01230-2). “This is an interesting multidisciplinary manuscript that addresses an important challenge in kinase inhibition. The manuscript describes a covalent inhibitor of the protein-protein interaction between Serine–arginine (SR) proteins and SR protein kinases (SRPKs). Targeting regulatory protein-protein interactions of kinases represents an alternative strategy to inhibit substrate phosphorylation by active site inhibition. SRs have been implicated in numerous conditions with invasive/metastatic processes in oncology of key importance. The manuscript is broad in scope exploring a range of covalent warheads on a peptide based orthosteric inhibitor of SRPK substrate binding. Ultimately an aryl-sulfonyl fluoride warhead was identified. A range of biochemical/biophysical methods, together with cell-based analyses are used to show the inhibitor acts on target with good potency and selectivity. One thing that I find interesting about covalent peptide-based inhibitors is that in contrast to conventional covalent small molecule inhibitors, it is necessary to generate a reagent that can covalently link to its target without self-reaction which given the range of functional groups in a peptide can be challenging to do!”

Editorial Board Member Dr Katrin Dulitz highlights *The isomer distribution of C*_*6*_*H*_*6*_ *products from the propargyl radical gas-phase recombination investigated by threshold-photoelectron spectroscopy* by Helgi Rafn Hrodmarsson et al. (10.1038/s42004-024-01239-7). “This recent study of the propargyl radical self reaction adds to our understanding of aromatic molecule formation in the interstellar medium.”

Chief Editor Dr Victoria Richards highlights *Conjugated bis(enaminones) as effective templates for rotaxane assembly and their post-synthetic modifications* by Syed S. Razi et al. (10.1038/s42004-024-01258-4). “This paper reports a clever templating strategy that results in the assembly of rotaxanes in high yields and with access to versatile structures through post-synthetic modifications. The team further showcase the utility of the method with assembly of a rotaxane for which control over molecular motion has been achieved.”

Editorial Board Member Prof. Wei Zhang highlights *On the interfacial phenomena at the Li*_*7*_*La*_*3*_*Zr*_*2*_*O*_*12*_ *(LLZO)/Li interface* by Kostiantyn V. Kravchyk et al. (10.1038/s42004-024-01350-9). “This Comment contributes significantly to the field of solid-state batteries by offering viable solutions to overcome the longstanding challenges at the LLZO/Li interface, marking a step forward in the quest for safer and more efficient energy storage solutions. An identification of specific chemical and physical changes occurring at the LLZO/Li interface during Li plating and stripping, enables providing insights into how these transformations impact electrochemical performance. They analyze the interfacial dynamics of enhancing lithium affinity, reducing interface resistance, and decreasing voltage polarization by the methods of cleaning the LLZO surface and utilizing an LLZO/Li interfacial layer. However, issues still arise with the formation of voids during the Li stripping process, which can be mitigated by applying pressure, enhancing Li diffusion within Li metal, and increasing the contact area between Li and LLZO.”

Editorial Board Member Dr François-Xavier Coudert highlights *Leveraging infrared spectroscopy for automated structure elucidation* by Marvin Alberts et al. (10.1038/s42004-024-01341-w). “The authors applied the power of transformer models to infrared (IR) spectroscopy, which is probably the most common and accessible analytical method in chemistry, but is limited in its interpretation to select functional groups with unambiguous signals. To train their machine learning (ML) model, they have combined both experimental and simulated IR spectra, highlighting the power of fine-tuning a pre-trained model when experimental data is scarce. This paper is very representative of advanced use of ML models in analytical and physical chemistry.”

Editorial Board Member Dr Per-Olof Syrén highlights *Mechanistic investigation of sustainable heme-inspired biocatalytic synthesis of cyclopropanes for challenging substrates* by Dongrun Ju et al. (10.1038/s42004-024-01371-4). “This paper demonstrates the power of quantum mechanical calculations to shed atomistic insight on reaction mechanisms underpinning enzyme catalysis, here with respect to the impact on non-natural amino acids, electron-withdrawing substituents and different macrocycles on the barrier for heme-based biosynthesis of cyclopropanes. The findings showcase that challenging electron-deficient acrylate substrates could act in cyclopropanation provided that the right macrocycle and non-natural axial haem ligand is provided, paving the way for expanded reaction scopes in biocatalytic transformations of high industrial relevance.”

Editorial Board Member Dr Felipe García highlights *The interplay between hydrogen bonds and stacking/T-type interactions in molecular cocrystals* by Aurora J. Cruz-Cabeza et al. (10.1038/s42004-024-01380-3). “Are hydrogen bonds the most important interaction in molecular cocrystals? Hydrogen bonds are traditionally seen as key to cocrystal formation, but analysis of 1:1 cocrystals in the Cambridge Structural Database reveals stacking and T-type interactions are just as crucial. Aromatic coformers dominate (84%), with only 20% of dimers relying solely on strong hydrogen bonds, while over 50% feature stacking and T-type contacts. Together, these interactions equally stabilise cocrystal lattices. This manuscript demonstrates that a balanced approach – optimising both hydrogen bonding and stacking/T-type interactions – may be beneficial for advanced cocrystal design in future crystal engineering studies when searching for novel cocrystals with new properties.”

Editorial Board Member Dr Satoshi Honda highlights *Modulating self-assembly and polymorph transitions in bisdendronized squaramides* by Sergi Bujosa et al. (10.1038/s42004-024-01391-0). “The team demonstrated that slight differences in the alkyl spacer length between the squaramide core and the two dendrons linked to it affect the association equilibrium. This study will provide important insights into the formation of structurally controlled aggregates and their morphological transformation.”

In addition to the above highlighted articles, we are pleased to include within our 2024 highlights a number of Review, Perspective and Comment pieces that contribute valuable opinions and thought leadership for the community. We hope you enjoy browsing this exciting Collection of *Communications Chemistry* content.

